# Supplementary material for: Pesticides and transgenerational inheritance of pathologies: Designing, analysing and reporting rodent studies
Source: PLoS One. 2020 Oct 1;15(10):e0228762. doi: 10.1371/journal.pone.0228762 (PMC7529424; doi:10.1371/journal.pone.0228762)
Supplement: S1 Appendix — (DOCX) [file pone.0228762.s001.docx]

**S1 Appendix**

The following adverse binary outcomes were used in the reanalyses of the WSU studies:

1. Puberty: this was separated into ‘early’ and ‘late’ problems in [4, 5] but is modelled as a single binary outcome here with ‘problem’ defined as either ‘early’ or ‘late’.

2. Testis: only measured in [5, 6].

3. Ovary: this was separated into ‘PFL’ and ‘PCO’ problems in [2] but is modelled as a single binary outcome here with ‘problem’ defined as either ‘PFL’ or ‘PCO’.

4. Prostate: measured in all five studies.

5. Kidney: measured in all five studies.

6. Lean: only measured in [4-6] and defined as the complement of obese in [4].

7. Obese: apparently measured in all five studies but with no positive observations and therefore treated as missing in [2].

It should be noted that there are inconsistencies across the datasets for control rats with the same ID. It was assumed that if a rat is defined as having an adverse outcome in one study but not in another then it is regarded as having an adverse outcome in all studies so, for example, rat AC6-3-6-5 (F3 control female), which has an ovary pathology in the glyphosate dataset but not in the atrazine dataset, is assumed to have a pathology in both. And if a measure is missing in one study but not in another then it is not missing throughout. These inconsistencies are concerning but are unlikely to have had any important effect on the results presented here.
