# Supplementary material for: Pesticides and transgenerational inheritance of pathologies: Designing, analysing and reporting rodent studies
Source: PLoS One. 2020 Oct 1;15(10):e0228762. doi: 10.1371/journal.pone.0228762 (PMC7529424; doi:10.1371/journal.pone.0228762)
Supplement: S2 Appendix — (DOCX) [file pone.0228762.s002.docx]

**S2 Appendix**

**Models for continuous outcomes**

A three-level hierarchical or nested model for a continuous outcome is:

$$y_{ijk}=\beta_{0}+v_{k}+u_{jk}+e_{ijk} 1(a)$$

where $y_{ijk}$ is the outcome of interest for each F2 rat; $i=1..n_{jk}$ indexes the F2 (level one) rats nested within F1 (level two) $j, j=1..J_{k}$ and F0 (level three) $k, k=1..K$; $v_{k} \sim N(0,\sigma_{v}^{2})$ is the random effect for F0 dams; $u_{jk} \sim N(0,\sigma_{u}^{2})$ is the random effect for F1 (within F0) and $e_{ijk} \sim N(0,\sigma_{e}^{2})$ with $\sigma_{e}^{2}$ the variance between F2 rats within both the F0 and F1 dams. Estimation proceeds as described in [28] and implemented in several statistical packages.

Litter effects can be represented by intra-litter correlations. There are two of these for a three level model: the correlation between two randomly selected F2 animals sharing the same grandmother but from different mothers = $\sigma_{v}^{2}/(\sigma_{v}^{2}+\sigma_{u}^{2}+\sigma_{e}^{2})$ and the correlation between two randomly selected animals sharing the same grandmother and mother = ${(\sigma}_{v}^{2}+\sigma_{u}^{2})/(\sigma_{v}^{2}+\sigma_{u}^{2}+\sigma_{e}^{2})$.

**Models for binary outcomes**

When the outcome is binary (all the outcomes from the WSU studies are binary) then the model corresponding to $1\left( a \right)$is now a generalised linear multilevel model:

$logit \left( \pi_{ijk} \right)= \beta_{0}+v_{k}+u_{jk};p_{ijk} \sim Bin\left( 1, \pi_{ijk} \right) 1(b)$

where $\pi_{ijk}$ is the probability of an adverse outcome and $p_{ijk}$ is the observed outcome with a binomial distribution. The random effects at levels two and three are defined as in$1(a)$. Although the estimation of models such as $1\left( b \right)$is computationally more complicated than it is for continuous outcomes, the methods are widely implemented. The intra-litter correlations are defined as for continuous outcomes except that $\sigma_{e}^{2}$is now fixed at $\pi^{2}/3$, the variance of the standard logistic distribution.

**Estimating treatment effects**

There two ways of estimating treatment effects with binary outcomes when there are litter effects. The first is just to extend model 1(b):

$logit \left( \pi_{ijk} \right)= \beta_{0}+{\sum_{l=1}^{5} \beta_{l}t_{lk}+ v}_{k}+u_{jk}$

where$t_{lk}$ are dummy (0/1) fixed effects for the five treatments in Tables 3 and 4. The estimates of β_l_ are known as cluster specific effects and give the differences between each treatment and the controls for rodents on the same points of the distributions of the two random effects.

Alternatively, we can estimate a model that omits the random effects but instead includes a term that represents the correlations between rodents within the same litter. These models are known as generalized estimating equations [29]; the estimates of β_l_ are known as population average effects and give the differences between each treatment and the controls for rodents averaged over all litters. The population average estimates are easier to interpret but the multilevel model provides an estimate of how much of the variation between clusters is accounted for by the treatments (and other variables).
