# Supplementary material for: Pesticides and transgenerational inheritance of pathologies: Designing, analysing and reporting rodent studies
Source: PLoS One. 2020 Oct 1;15(10):e0228762. doi: 10.1371/journal.pone.0228762 (PMC7529424; doi:10.1371/journal.pone.0228762)
Supplement: S1 Table — (DOCX) [file pone.0228762.s003.docx]

**S1 Table. Number of F0 Females by Study, Group and Generation**

| Study | Year | Pesticide | Group^(1)^ | F1 | F2 | F3 |
| --- | --- | --- | --- | --- | --- | --- |
| WSU1 | 2012 | Permethrin+DEET | T | 4 | n.a.^(2)^ | 3 |
|  |  |  | C | 7 | n.a. | 5 |
| WSU2 | 2014 | Methoxychlor | T | 4 | n.a.^(2)^ | 4 |
|  |  |  | C | 7 | n.a. | 6 |
| WSU3 | 2017 | Atrazine | T | 5 | 5 | 5 |
|  |  |  | C | 13 | 6 | 5 |
| WSU4 | 2018 | Vinclozolin | T | 15^(3)^ | 10^(4)^ | 9^(4)^ |
|  |  |  | C | 12^(5)^ | 8 | 5 |
| WSU5 | 2019 | Glyphosate | T | 6 | 5 | 5 |
|  |  |  | C | 14 | 8^(6)^ | 7^(6)^ |

Notes

^(1)^ Treatment (T) or control (C).

^(2)^ No measurements at F2.

^(3)^ n = 10 in article but n = 15 in dataset.

^(4)^ Includes five F0 not used at F1.

^(5)^ Five of controls also used in WSU3 and WSU5.

^(6)^ Includes one F0 not used at F1.
