# Supplementary material for: Pesticides and transgenerational inheritance of pathologies: Designing, analysing and reporting rodent studies
Source: PLoS One. 2020 Oct 1;15(10):e0228762. doi: 10.1371/journal.pone.0228762 (PMC7529424; doi:10.1371/journal.pone.0228762)
Supplement: Attachment — Submitted filename: PLOS_ref2_rev.docx [file pone.0228762.s007.docx]

Reviewer #2: 1) The author has expressed a severe bias against epigenetic inheritance and studies involved in the analysis of environmental toxicants such as glyphosate. A scholarly approach and journal such as Plos One requires an unbiased presentation of the data and discussion of the topic and study. An example is the first sentence in the Abstract that made a generalized derogatory statement against the field of epigenetic inheritance and transgenerational studies. With over 3000 publications on the topic and over 100 different laboratories reporting studies in nearly 20 different species, to make a general statement condemning the field demonstrates a bias that is not appropriate in a scholarly publication. ABSTRACT MODIFIED AS SUGGESTED BY THE EDITOR

Other biased statements that need to be removed include page 2, lines 1-3, 18-20, page 3 lines 17-19, page 4 lines 1-2, 4-5, 21-23, page 5 lines 5-6, 8-9, page 15 lines 12-13, page 16 lines 6-13.

I DISAGREE THAT THESE ARE BIASED STATEMENTS.

In addition, the author in assessing the studies and applying his arguments makes assumptions and subjective statements regarding the studies that are not based on the original studies described information. This indicates experimental design issues such as page 4 lines 2, 15, page 5 lines 1-4, page 6 lines 9, 10, 15-17, page 7 lines 2-5, 8-9, page 15 lines 12-13. The author generally makes overarching comments about the field and transgenerational studies, rather than the studies investigated. The bias against studies investigating pesticide impacts, such as those with glyphosate, are not appropriate and question the potential conflicts of interest of the author and analysis. A thorough revision removing these biases is required.

THERE WERE NO CONFLICTS OF INTEREST AS STATED IN MY SUBMISSION (AND SUBSEQUENTLY) AND IT IS WRONG OF THIS REVIEWER TO ASSERT OTHERWISE. I HAVE NO BIAS FOR OR AGAINST PESTICIDES, ONLY A WISH TO SEE RISKS PROPERLY ASSESSED.

2) A major deficiency is the author developed an approach to assess litter effects and used data for kidney, testis, ovary or prostate in the supplement S2, but did not report data or reanalyze results of litter effects on any of these pathologies. The assumption is that no litter effects were observed, however, the author needs to more fully disclose all data from the analysis. A negative effect of litter on these pathologies should be reported to put the current study into a better perspective, and fully disclose the results for the study. All data analyses need to be reported, both positive and negative.

THIS IS SIMPLY UNTRUE – LITTER EFFECTS FOR ALL THE OUTCOMES ARE REPORTED IN TABLE 1.

3) A major experimental flaw in the analysis is the combination of males and females, as well as the combination of all treatments into one data set used for the analysis. This disregards critical biological issues in male and female responses to specific exposures and differences in disease etiology between the males and females for specific pathologies. The control of puberty and metabolic disease in males and females are often opposite, as well as are the actions of different endocrine disruptors on pathology. Estrogenic compounds promote a premature puberty in females and delayed puberty in males, while androgenic effects are opposite. To combine males and females as an experimental unit negates the biological differences and complicates the disease correlations with litters sought. The combination of some estrogenic exposure and some androgenic exposures and others that are both, also negates the different biological effects of the exposures by combining data. Therefore, the experimental design may generate observations that are distinct from the experiments and randomly derived. To combine these and assess impacts is questionable. What is requested is that the author performs the same analysis on the isolated data sets without the combination of the sex or exposure. This would assess the potential litter effect within an experiment and not bias it by combining sex and exposure. This additional analysis will be useful to determine if combined data versus individual study data without males and females combined, provides distinct observations. The potential litter effects and different approach to the analysis will then provide a more biologically appropriate assessment of the study objective.

THESE COMMENTS ARE UNDERSTANDABLE FROM A BIOLOGICAL STANDPOINT AND I HAVE ADDED SOME SENTENCES TO RECOGNISE THIS. THIS PAPER IS, HOWEVER, CONCERNED WITH METHODOLOGY AND, AS EXPLAINED IN THE TEXT, IT IS NECESSARY TO COMBINE THE DATASETS (AND THE SEXES WHERE APPROPRIATE) IN ORDER TO IMPROVE THE ESTIMATION OF THE LITTER EFFECTS. THERE IS NO OBVIOUS REASON FOR LITTER EFFECTS TO BE DIFFERENT FOR MALES AND FEMALES. IN MY VIEW, COMBINING THE DATASETS IS A STRENGTH OF THE PAPER WHICH DOES NOT COMPROMISE THE VALIDITY OF THE ANALYSES.

4) Many of the exposure studies and pathology were unique to a specific exposure and distinct from other studies. For example, the atrazine exposure promotes a lean phenotype and none of the other exposures do so, while instead many of the others promote an obese phenotype. To combine different exposures and males and females complicates any such analysis. The Tables presented did not provide sufficient information to determine if isolated treatment sets were used, but did appear to combine treatments and males and females. Again, a truly isolated analysis for a specific treatment and exposure is needed to compare the combined data presented now in the manuscript.

SEE ABOVE. THE “EFFECTS” OF THE DIFFERENT TREATMENTS ARE ASSESSED (TABLES 3 AND 4) BUT, IN ORDER NOT TO OVERLOAD THE PAPER, ONLY FOR TWO OF THE OUTCOMES AS EXPLAINED.

5) The puberty and obesity pathologies are not able to be assessed in males and females combined in the analysis as presented in the current study. Since both pathologies are distinct and induced differently by the same exposure, combination of the sexes for analysis is difficult to both interpret and make any biological inferences. New data analysis is needed with the isolated sexes and exposures to compare with the combined data presented.

THIS JUST REPEATS POINT 3.

6) As described for the previous papers’ studies, the F0 generation females were randomly selected for treatments. The author makes the statement that randomization was compromised for these studies because there is an imbalance between the number of controls and treated animals. This seems a marked overstatement, as the Fisher’s Exact test handles this well.

RANDOM SELECTION AND RANDOMISATION ARE DIFFERENT ISSUES. RANDOMISATION IS A RESEARCH PROCESS AND ANY POST-HOC TEST OF GROUP SIMILARITY IS IRRELEVANT. IT IS CLEAR THAT RANDOM ASSIGNMENT OF RATS TO TREATMENTS WAS NOT CARRIED OUT.

7) The justification for combining the various studies into one analysis seems weak. The Permethrin+DEET study had its own controls and published many years separated from the others. The Methoxychlor study had its own controls and was a few years separated from the others. The Vinclozolin and Atrazine studies shared controls. The Glyphosate study shared some but not all controls with the Vinclozolin and Atrazine studies, and had some of its own. It would improve the manuscript to present the results of analyzing these studies separately, as well as combined.

AGAIN, SAME POINT AS EARLIER.

8) According to the previous papers studies, the F0 generation females were randomly selected and no pathology on the females was performed, so the comments to the contrary by the author should be removed from the manuscript. In addition, the statement on page 7 that an imbalance in treated and control n-value is a failure to randomize is misleading and inaccurate statement. Only three of the studies appear to have shared controls, so this is not a good justification to combine the data for the analysis as suggested by the author. The final statement in the Appendix S1 that common controls were used and information is missing is a presumption by the author that is not appropriate.

SEE ABOVE

9) The Table legends need significantly more information to help interpret the numerical values presented, clarify ranges presented and state males and females combined, as well as treatments. For Table 1, all data appear to be combined for a specific pathology. Due to the male versus female and distinct actions of exposures, misleading data will be obtained when correlations are made for disease. For Table 2, details in the legend are needed on what the different value ranges represent. Tables 3 and 4 legends need to help clarify and define values and ranges presented. Insufficient information is available to have an expert or non-expert interpret what is reported.

AS REQUESTED BY REFEREE 1, ADDITIONAL EXPLANATORY MATERIAL HAS BEEN ADDED.

10) The Conclusion presented has a bias expressed that is not linked to the data presented or analysis. This conclusion needs to be removed for a more objective and scholarly reflection on the current study analysis and include limitations of the analysis (e.g. combining data sets) that impact the interpretations.

CONCLUSION REWRITTEN

This study presents some good ideas on how to account for ancestral litter relatedness in analyzing data of this type, and how to improve reporting of data including breeding records. If the deficiencies listed above are addressed, this will be a useful addition to the literature.
